# Unusual Metastasis of Papillary Thyroid Cancer to the Thoracic Spine: A Case Report, New Surgical Management Proposal, and Review of the Literature

**DOI:** 10.7759/cureus.1132

**Published:** 2017-04-03

**Authors:** Ariel Takayanagi, Omid Hariri, Hammad Ghanchi, Dan E Miulli, Javed Siddiqi, Frank Vrionis, Farbod Asgarzadie

**Affiliations:** 1 Department of Neurosurgery, Riverside University Health System Medical Center, Moreno Valley, California, United States; 2 Department of Neurosurgery, Riverside University Health System-Medical Center, Moreno Valley, California, United States; 3 Department of Neurological Surgery, Marcus Neuroscience Institute, Boca Raton, Florida, United States; 4 Department of Neurosurgery, Kaiser Permanente Fontana Medical Center, Fontana, California, United States

**Keywords:** papillary thyroid carcinoma, thyroid follicular carcinoma, spinal metastases, metastases of unknown origin, spinal cord compression, embolization, preoperativve, hypervascular, thoracic, spine surgery

## Abstract

Papillary thyroid carcinoma (PTC) is significantly more common than follicular thyroid carcinoma (FTC), yet FTC has a much higher tendency to metastasize to the spinal column. We present a rare case of a metastatic thoracic spinal column lesion originating from the PTC. Thyroid carcinoma is known to be highly vascular with a significant tendency to hemorrhage during surgical resection. This increased tendency to hemorrhage leads to unanticipated intraoperative risks when the type of cancer is not diagnosed before surgical resection. Complications related to intraoperative bleeding can be prevented by visualization using angiography and preoperative embolization. The type of cancer is ideally diagnosed before tumor resection either by the standard metastatic workup or histologically after the biopsy. However, limitations exist in these methods, therefore, hypervascular tumors such as metastatic thyroid cancer can go undiagnosed until after surgical resection. In addition to our case report, we present a review of the literature regarding diagnostic and treatment strategies for hypervascular thyroid tumors and propose a new algorithm for the surgical management of spinal tumors with an unknown origin for optimization of preoperative and perioperative care.

## Introduction

The bone is the second most common location to which thyroid cancer is known to metastasize, affecting three percent of patients with thyroid cancer [1]. Although papillary thyroid carcinoma (PTC) accounts for 75% of thyroid cancer while follicular thyroid carcinoma (FTC) accounts for 15% to 20% of thyroid cancer, FTC is much more likely to be found in the spine than PTC (7% to 28% and 1.4% to 7%, respectively) [2]. This is likely due to the tendency of FTC to spread hematogenously, while PTC tends to spread through the lymphatic system. We present a rare case of a metastatic thoracic spinal lesion originating from the PTC and a brief review of the literature regarding the surgical management of metastatic spinal tumors originating from the thyroid gland. We propose a new algorithm for the management of spinal tumors with unknown tissue origins.

## Case presentation

A 52-year-old woman with history of hypertension presented to the emergency department with a two-month history of ataxia, mid-thoracic back pain, and paresthesias in the distal lower extremities bilaterally. She reported that her symptoms had become progressively worse over the past two weeks, and she began experiencing urinary incontinence one week prior to admission. On physical examination, we noted significant bilateral clonus in her lower extremities, and strength of 4+/5 in her lower extremities bilaterally. Moreover, she required a Foley catheter placement given her significant urinary incontinence.

**Figure 1:**
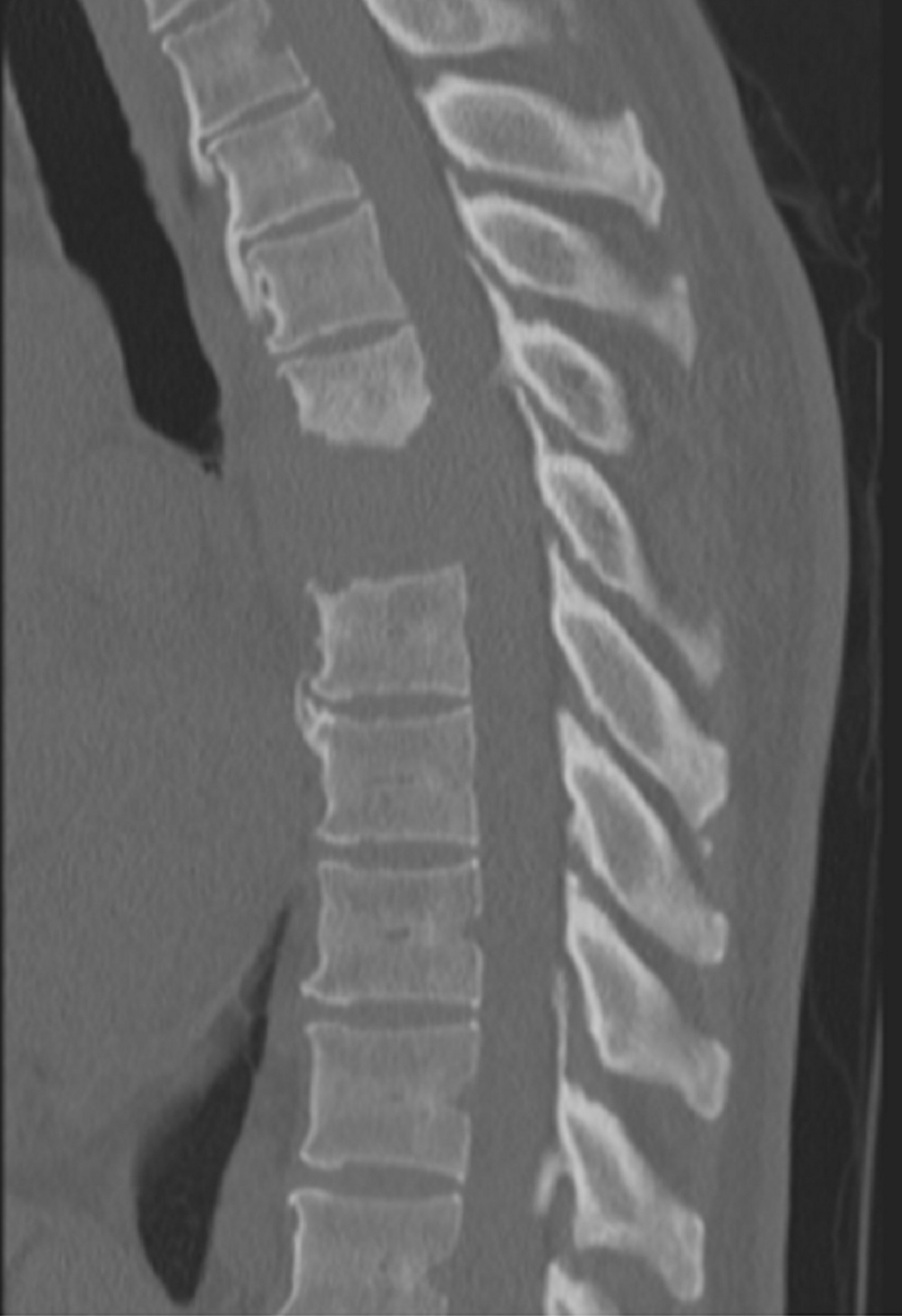
Sagittal CT of the thoracic spine Sagittal computed tomography (CT) of the thoracic spine demonstrating a lytic bone lesion primarily involving the T7 vertebra with some extension cranially into T6 and mild caudal extension into the superior endplate of T8.

**Figure 2:**
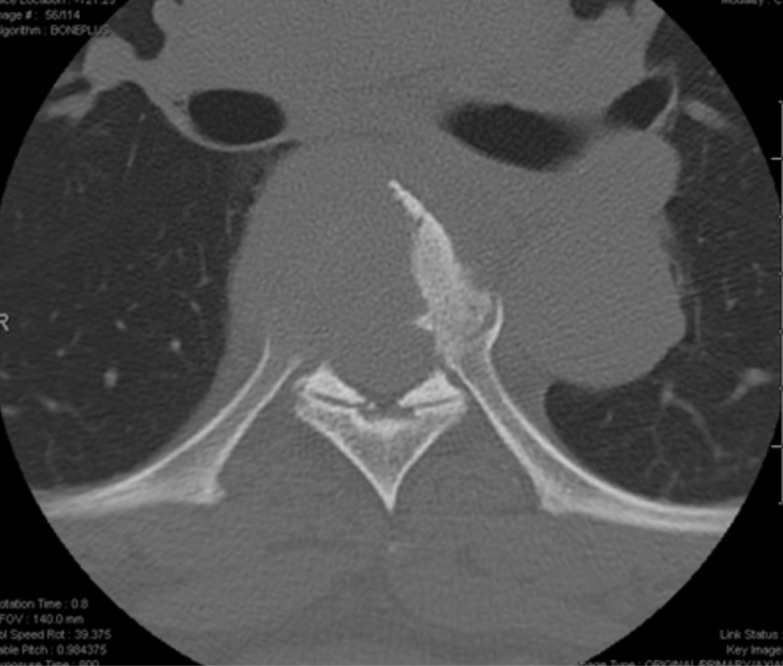
Axial CT of T7 Axial thoracic spine computed tomography (CT) demonstrating a lytic bone lesion at the level of T7.

**Figure 3:**
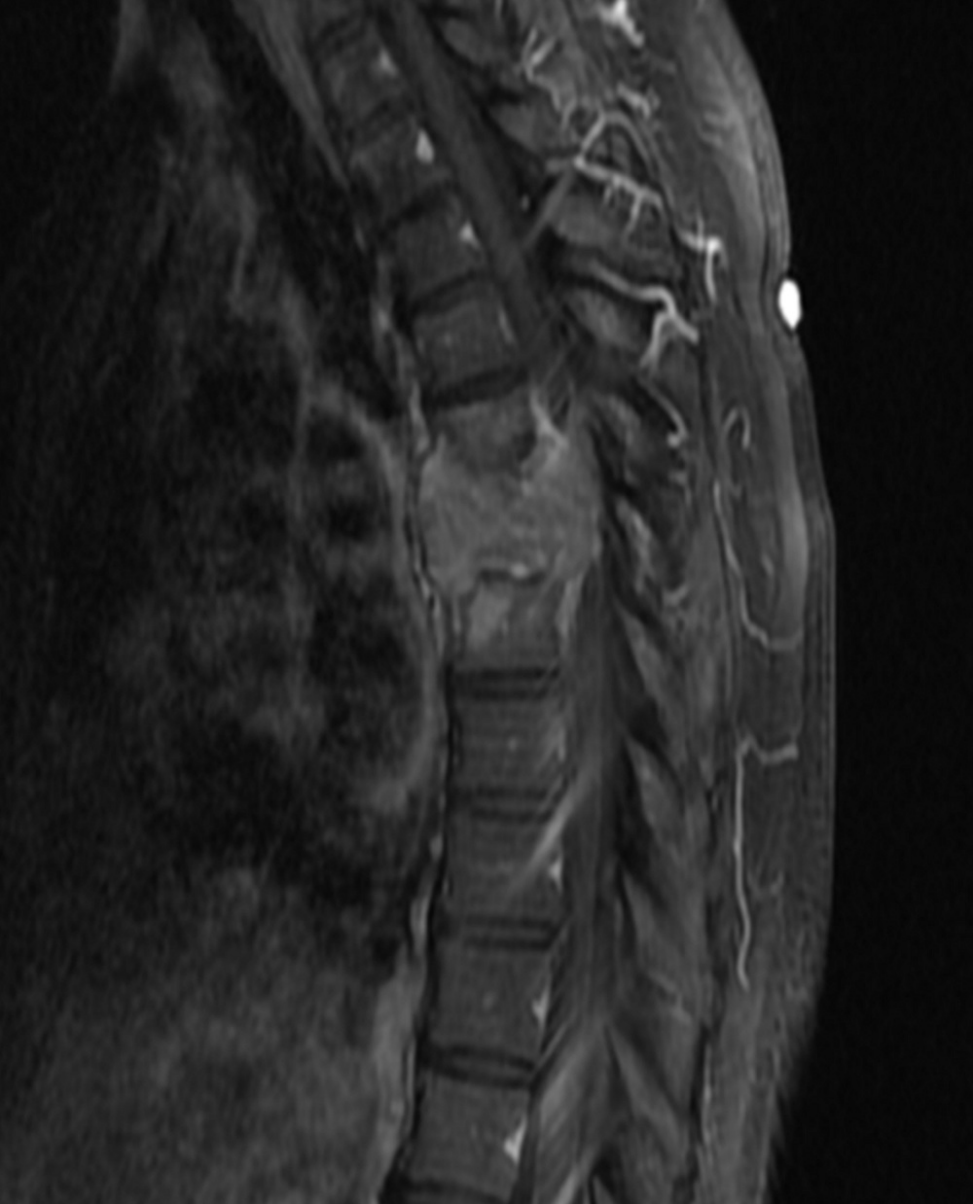
Sagittal T1 weighted MRI of thoracic spine with contrast Sagittal T1 weighted magnetic resonance imaging (MRI) of the thoracic spine with contrast demonstrating a homogeneously enhancing mass lesion in the T7 vertebral body invading superiorly and inferiorly into the intervertebral discs.

**Figure 4:**
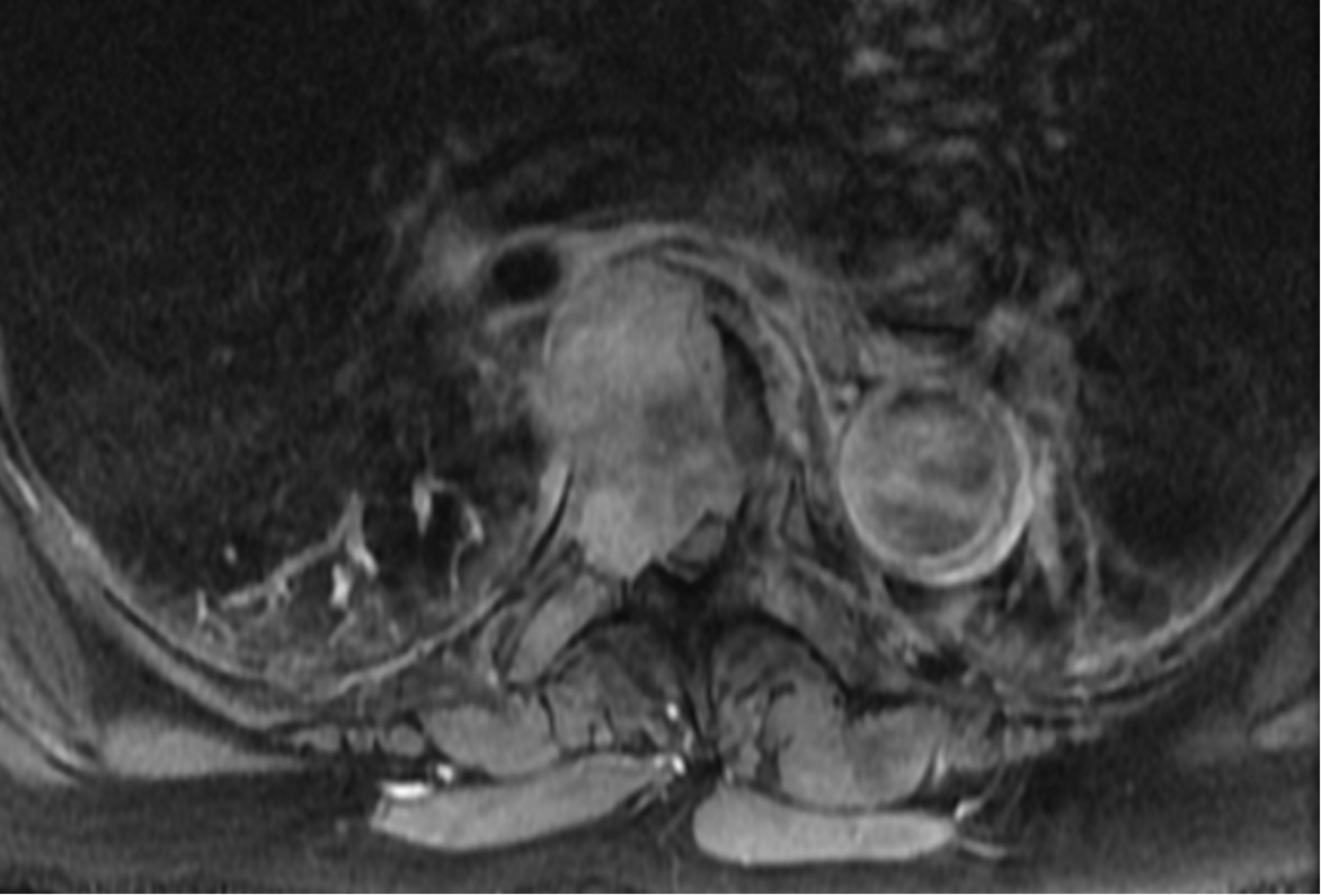
Axial T1 weighted MRI of the thoracic spine with contrast Axial T1 weighted magnetic resonance imaging (MRI) of the thoracic spine with contrast at the level of T7 vertebral body shows the mass extending into the right pedicle, invading into the spinal canal, and displacing the spinal cord to the left.

Computed tomography (CT) and magnetic resonance imaging (MRI) scans of the thoracic and lumbar spine showed a 4 cm lytic lesion of the T7 vertebral body producing a mass effect on the spinal cord (Figures 1-4). Imaging of the lumbar spine did not show any evidence of metastatic disease. A nuclear bone scan displayed lesions of T6 and T7 vertebral bodies and no other lesions were present in the spine or other parts of the skeletal system. The location of the mass corresponded with the neurologic exam; therefore, treatment was focused on this region. Evaluation of the cervical spine and the brain and the positron emission tomography (PET) was planned for after the surgical procedure.

At this time, common types of cancer to metastasize to the spine were high in the differential diagnosis, including lung, breast, and kidney as well as less common types such as thyroid. CT scans of her chest, abdomen, and pelvis were negative for other metastases, and the results of laboratory studies were within normal limits, including a thyroid panel (thyroid stimulating hormone: 0.4, free thyroxine: 0.8) which was low-to-normal.

**Figure 5:**
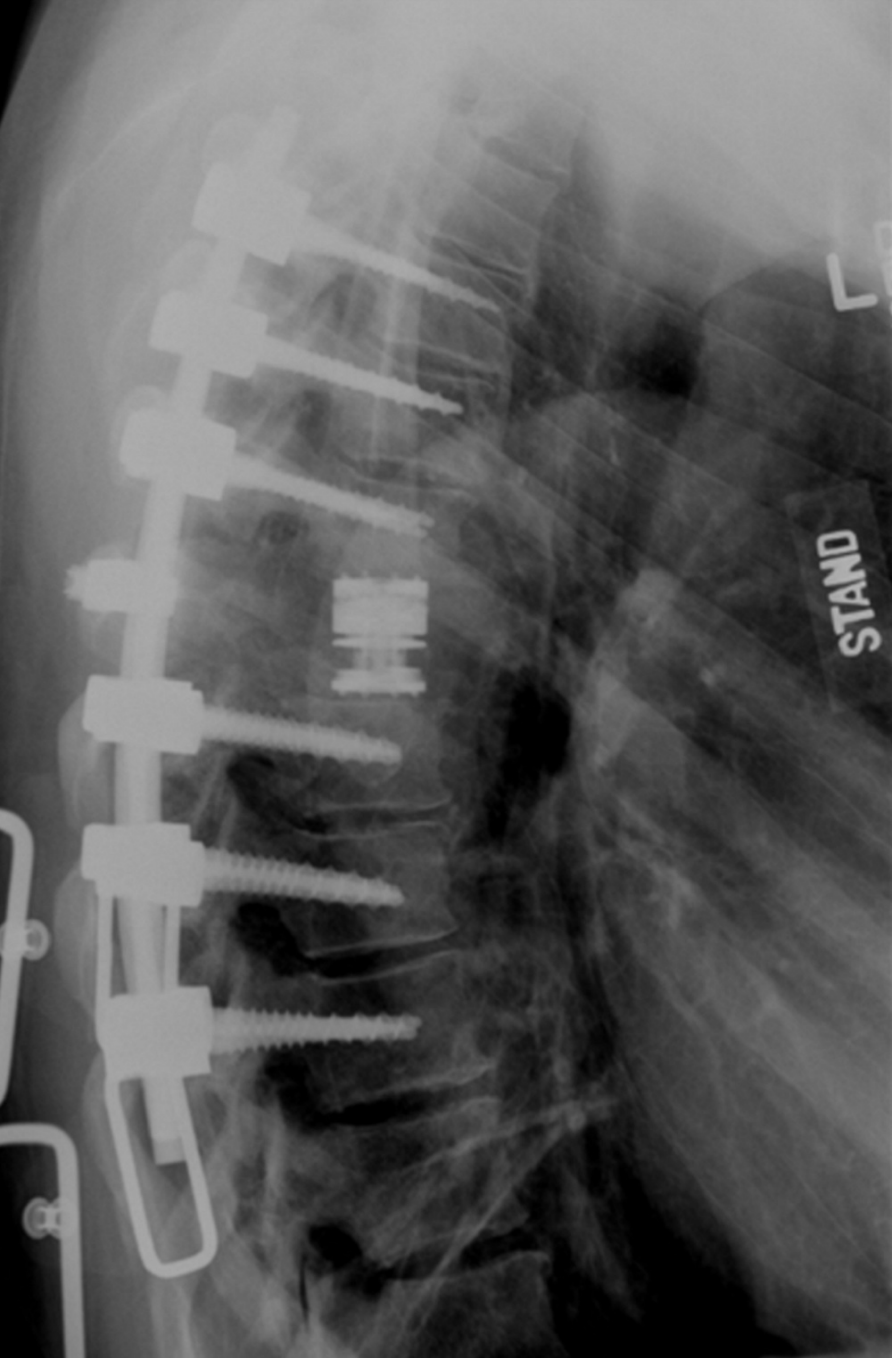
Postoperative upright lateral thoracic x-ray Screws T4 through T10, skipping T7. Expandable cage and a single cross-link at T7. Maintenance of the patient’s anatomical kyphotic curve is demonstrated.

Given the acute neurological decline, the patient was placed on intravenous (IV) corticosteroids to reduce edema of the spinal cord. Moreover, the therapeutic goal for this patient was spinal cord decompression and stabilization. Unfortunately, the patient declined the option of undergoing percutaneous biopsy of the vertebral lesion but consented to surgical resection. A lateral extracavitary approach from the right side was used, and stereotactic guidance aided the placement of pedicle screws from T4 through T10, excluding T7. Laminectomies of T6 and T7 revealed a highly vascular mass displacing the spinal cord posteriorly and to the left. The mass was resected in a piecemeal fashion, and complete vertebrectomies were achieved. The right T7 nerve root was removed to facilitate placement of an expandable interbody cage filled with allograft from T6 to T8. Figure 5 shows a postoperative lateral upright X-ray.

Postoperatively, the patient’s neurological exam results showed improvement with motor strength improving to 5/5 in the bilateral lower extremities, and she reported a reduction in back pain. The Foley catheter was removed on postoperative day 2, and an ultrasound of the bladder showed no urinary retention. Once the pathology consultant confirmed the diagnosis as PTC, an otolaryngologist and radiation oncologist were consulted for further treatment of the thyroid neoplasm, which was confirmed on CT soft tissue of the neck. The patient was discharged from the hospital. After the patient was discharged from the hospital, the patient did not follow up with the neurosurgery clinic due to having a health care plan that was outside of the hospital’s network. 

## Discussion

The workup of a tumor of unknown origin ideally includes a biopsy, but it is often not feasible due to the time needed to schedule a biopsy and receive a final histological diagnosis. In addition, biopsies of spinal tumors can often be nondiagnostic. In a study comparing the diagnostic usefulness of CT scans of the chest, abdomen, and pelvis, along with CT-guided biopsy and laboratory testing for the diagnosis of spinal metastases of unknown origin, a CT-guided biopsy was nondiagnostic in 15 out of 27 patients (55%) with solid tumors [3].

A diagnostic workup is usually faster than a biopsy and includes CT scans of the chest, abdomen, and pelvis as well as immunoelectrophoresis and bone scans. If the patient does not present as an emergency, administering steroids will typically allow for the 24 hours needed for the workup. The workup will typically cover potential lung, gastrointestinal, gynecologic, and urologic cancer as well as breast and prostate cancer and myeloma.

In rare cases of adenocarcinoma, the primary tumor can be very small and elude detection. Most melanoma metastases are preceded by a cutaneous primary tumor and are less likely to present as a tumor of unknown origin. Primary sarcoma and lymphoma may also need to be considered, and the diagnosis is typically made with a biopsy. A biopsy is especially important in primary spine tumors including chordoma, chondrosarcoma, and synovial sarcoma because these may be best resected en bloc [4].

Thyroid cancer, in particular, has risks associated with resection in the absence of angiography and embolization and thyroid tests are not included in the standard imaging studies for metastatic spinal disease. Yet, over one-third of the patients with spinal metastases from thyroid cancer will present initially with spinal symptoms [1], as did the patient in the present case.

Thyroid metastases are known to be hypervascular with large arterial feeders as well as rich capillary beds [5]. Diagnosing vertebral metastases from hypervascular tumors such as thyroid metastases is essential to optimize surgical planning and treatment.

Selective embolization of hypervascular tumors may be done preoperatively to decrease the risk of complications related to extensive blood loss during surgery. Preoperative embolization of hypervascular bone metastases has been shown to significantly decrease intraoperative blood loss (0.90 versus 1.77 liters; p = .002), packed red blood cell transfusion volume (2.15 versus 3.56 units; p = 0.020), and operative time (3.13 versus 3.91 hours; p < 0.001) [6]. With embolization, patients will likely experience fewer risks associated with blood transfusions and prolonged time under anesthesia. The extent to which the tumor is embolized also significantly affects the volume of blood loss during surgical removal of metastatic spinal tumors. In a retrospective study of 66 posterior decompression procedures for 26 renal cell and 39 thyroid carcinoma spinal metastases, tumors with complete embolization bled significantly less during surgery compared to those with incomplete embolization (809 ± 835, 1210 ± 904, respectively; p = 0.03) [7].

In addition to minimizing the volume of blood loss, the need for blood transfusions, and the operative time, selective embolization may also allow for more aggressive resection. Hess, et al. [8] compared 17 patients with spinal tumors originating from thyroid carcinoma (n = 2), renal cell carcinoma (n = 13), or adenocarcinoma (n = 2) of unknown origin who underwent preoperative embolization to 17 patients who did not undergo embolization. Consistent with previously stated studies, patients who underwent angiography and embolization were reported to have significantly less blood loss during surgery (2088 mL versus 3880 mL; p = 0.024). However, two patients from the non-embolized group who had spinal tumors with unknown primary cancer experienced such extensive blood loss that the operation was aborted before the planned tumor resection could be completed [8]. When the tumor type is known before the operation, such complications can be prevented by embolization. In addition, better resection with embolization may also occur due to the enhanced ability to visualize the tumor [5].

While the degree of vascularity can sometimes be detected on an MRI, the sensitivity is low. Prabhu, et al. [9] found that of 17 tumors thought to be highly vascular on the MRI, 13 were truly hypervascular on angiography. Of 19 patients found to have low to moderate vascularity on the MRI, 15 were found to be highly vascular with angiography. These results indicate that MRI findings are not adequate to rule out the possibility of high vascularity in metastatic spinal neoplasms [9].

Moreover, in cases of lower thoracic spinal tumors, angiography is an important preoperative diagnostic tool to better assess the vasculature of the spinal cord. Visualizing the vasculature decreases the unfortunate event of injuring the artery of Adamkiewicz which will lead to paralysis.

While angiography and embolization are important considerations in a non-acute setting, surgical decompression and stabilization are priorities in patients with acute neurological deterioration. Angiography with the necessary anesthesia in this setting may result in decreased blood pressure and cord perfusion, while no decompression is achieved. When faced with acute neurologic decompensation with severe epidural spinal cord compression and a risk of imminent catastrophic spinal cord infarction, it is appropriate to proceed with emergent decompression and fusion.

Conversely, in a patient with advanced metastatic disease and spinal metastases, whose predominant symptom is mechanical pain with no significant signs of neurological deficits, minimally invasive surgery can be performed as a palliative measure. Techniques include vertebral augmentation in possible combination with percutaneous pedicle screw placement in the setting of spinal instability. Moreover, radiofrequency ablation in cases with large tumors can be applied in combination with vertebral augmentation. A recently described technique called spinal laser interstitial thermal therapy involves thermal ablation of the tumor adjacent to the dura, decompressing the spinal cord while creating a safe margin around the spinal cord for high-dose radiotherapy. The short recovery time for this technique allows for a prompt resumption of systemic therapy. [10]

**Figure 6:**
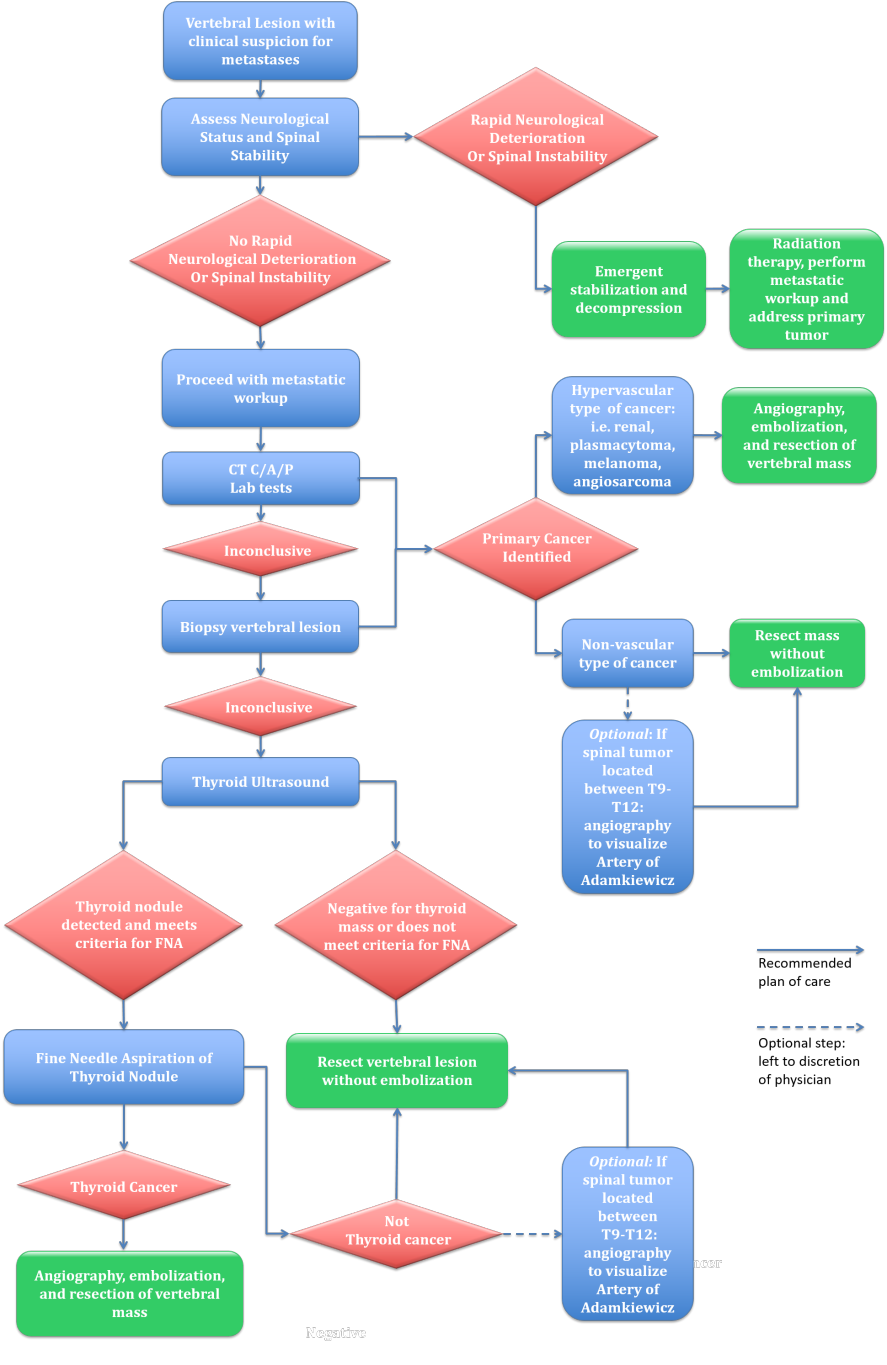
Proposed algorithm for vertebral metastases suspicious for metastases Computed tomography of chest, abdomen, pelvis (CT C/A/P).

In a non-emergent setting, we propose that a thyroid ultrasound should be done in patients when laboratory studies and CT scans of the chest, abdomen, and pelvis, along with percutaneous biopsies of the spinal lesion, are all inconclusive. Thyroid ultrasound is a noninvasive, inexpensive, and widely available test that can lead to the diagnosis of thyroid cancer and, therefore, determine the need for embolization prior to surgery. Although larger studies are necessary to validate this algorithm, we believe the thyroid ultrasound is an easy addition to the metastatic workup of spinal bony lesions that has the potential to significantly change management, improve surgical efficiency and decrease risk (Figure 6). 

## Conclusions

When thyroid neoplasms metastasize to the vertebral column, the risk of intraoperative hemorrhage should be minimized. When the primary origin of cancer is not determined after the standard metastatic workup, further steps should be taken for diagnosis before resection. We propose that thyroid ultrasound should be added to the metastatic workup when CT scans of the chest, abdomen, and pelvis, along with laboratory studies and percutaneous vertebral biopsy, do not lead to a diagnosis. Adding this imaging modality has the potential to improve both surgical efficacy and safety. However, further studies are required to validate the application of ultrasound in this setting.

When thyroid carcinoma is diagnosed as the source, embolization can be done before surgery to minimize intraoperative blood loss, decrease the operative time, and provide an opportunity for improved visualization of the mass. This enables a more robust resection. Because thyroid cancer is prone to intraoperative complications, knowing the diagnosis before surgery will likely lead to a safer treatment due to better preoperative planning.
